# Inflammatory and Oxidative Stress Markers Related to Adherence to the Mediterranean Diet in Patients with Metabolic Syndrome

**DOI:** 10.3390/antiox11050901

**Published:** 2022-05-01

**Authors:** Maria Magdalena Quetglas-Llabrés, Margalida Monserrat-Mesquida, Cristina Bouzas, Cristina Gómez, David Mateos, Tomàs Ripoll-Vera, Josep A. Tur, Antoni Sureda

**Affiliations:** 1 Research Group in Community Nutrition and Oxidative Stress, University of the Balearic Islands-IUNICS, 07122 Palma de Mallorca, Spain; m.quetglas@uib.es (M.M.Q.-L.); margalida.monserrat@uib.es (M.M.-M.); cristina.bouzas@uib.es (C.B.); cristina.gomez@ssib.es (C.G.); davidfrom13@gmail.com (D.M.); tripoll@hsll.es (T.R.-V.); antoni.sureda@uib.es (A.S.); 2 Health Research Institute of Balearic Islands (IdISBa), 07120 Palma de Mallorca, Spain; 3 CIBER Fisiopatología de la Obesidad y Nutrición (CIBEROBN), Instituto de Salud Carlos III (ISCIII), 28029 Madrid, Spain; 4 Clinical Analysis Service, University Hospital Son Espases, 07198 Palma de Mallorca, Spain; 5 Cardiology Service, University Hospital Son Llàtzer, 07010 Palma de Mallorca, Spain

**Keywords:** metabolic syndrome, cardiovascular risk, Mediterranean diet, oxidative stress, inflammation

## Abstract

Metabolic syndrome (MetS) is characterized by increased pro-oxidative stress and a pro-inflammatory state. Several studies emphasized the protective effect of the Mediterranean dietary pattern (MDP). To assess the oxidative and inflammatory state according to the adherence to MDP using biomarkers in patients with MetS. Antioxidant and pro-inflammatory biomarkers were determined in plasma, peripheral blood mononuclear cells (PBMCs), and neutrophils of adults (aged 55–75 years old; 60% women) with MetS living in Mallorca (Spain). Anthropometrics, dietary intake by a validated semi-quantitative 143-item food frequency questionnaire, and a Dietary Inflammatory Index were measured. Patients with low adherence to MDP showed higher levels of glycated haemoglobin A1c and triglycerides, and lower levels of HDL cholesterol. Plasma levels of interleukin-1β, IL-6, IL-15, tumour necrosis factor α, xanthine oxidase, and ghrelin, and activities of superoxide dismutase, and myeloperoxidase were higher in subjects with low adherence to the MDP. Reactive oxygen species production in PBMCs and neutrophils stimulated with lipopolysaccharide was higher in participants with low adherence to the MDP. Patients with MetS and higher adherence to the MDP showed less altered anthropometric parameters, blood biochemical profile, and better oxidative and inflammatory status.

## 1. Introduction

Metabolic syndrome (MetS) is a cluster of risk factors including abdominal obesity, hypertension, dyslipidaemia, and raised fasting glucose [1]. This pathology has been associated with a higher rate of suffering from type 2 diabetes mellitus (T2DM), cardiovascular diseases (CVD), and cancer worldwide [2]. The prevalence of MetS has increased rapidly throughout the population in recent years, in the same way that the prevalence of T2DM and obesity are becoming a major public health problem [3]. It has been estimated that during the last 15 years, MetS prevalence increased from 34.3% to 38.5% [4]. The etiology of MetS, although largely unknown, is thought to reside in a complex interaction between genetic predisposition and metabolic and environmental factors [5].

To date, the best strategy to prevent the onset and progression of MetS is to adopt a balanced diet along with a healthy lifestyle [6]. In particular, several studies emphasized the protective effect associated with the Mediterranean diet pattern (MDP) on the different components of MetS [6,7]. However, its healthy properties cannot be specifically attributed to any particular nutrient, they have to be extended to the entire meal pattern and lifestyle [8]. In this sense, the protective role of the MDP derives from the high presence of bioactive compounds such as antioxidants, healthy fatty acids, fibre, and phytosterols mainly found in vegetables, fruits, legumes, virgin olive oil, fish, nuts, red wine, and probiotics derived from fermented foods [9,10]. Moreover, a moderate-intensity physical activity practiced regularly together with a healthy and energy-restricted dietary pattern are key strategies to prevent or improve metabolic disorders [11,12].

A feature of MetS is its relationship with a pro-oxidative and pro-inflammatory state [13]. The pathogenesis of MetS is associated with a rise in reactive oxygen (ROS) and nitrogen (RNS) species combined with a decreased antioxidant capacity [14]. This state results in an unbalanced redox and a low-grade inflammatory state directly related to the accumulation of visceral fat [13]. These reactive species, such as superoxide anion (O_2_^•−^), hydrogen peroxide (H_2_O_2_), or nitric oxide (^•^NO) when found in the normal physiological concentrations, act as cellular messengers driving cellular activities. However, when their concentrations are excessive, they can oxidize lipids, proteins, and nucleic acids leading to oxidative damage [14]. To neutralize this excessive production the organisms possess an antioxidant system that includes antioxidant enzymes such as catalase and superoxide dismutase (SOD) and non-enzymatic molecules such as polyphenols, vitamins, or glutathione (GSH) [15]. The increased oxidative stress favours the appearance of inflammation which, in turn, can alter the vascular function and lead to vascular disease [16].

The characteristic chronic low-grade inflammation present in MetS patients is associated with the expansion of adipose tissue [17]. Adipose tissue, in addition to being an energy storage organ, has the ability to secrete hormones and inflammatory mediators such as leptin, resistin, interferon- γ (IFN-γ), tumour necrosis factor α (TNFα), and interleukin (IL)-6, involved in energy homeostasis, insulin sensitivity and immune function [18,19]. Macrophages are the most abundant leukocyte population in the expansion of adipose tissue and, are also responsible to produce pro-inflammatory cytokines, such as TNFα, IL-6, and IL-1β, which promote insulin resistance [20]. Moreover, secretion of MCP-1 by adipocytes promotes increased macrophage recruitment to adipose tissue [21]. In addition, IL-15 is an inflammatory cytokine secreted by many cell types, contributing to inflammation in adipose tissues in obesity-associated MetS [22]. Obesity changes the production of leptin and ghrelin, two hormones secreted by adipose tissue and gastric tissue, respectively, which are involved in appetite regulation and the control of body weight [23].

Considering the antioxidant and anti-inflammatory properties of MDP, the main goal of this study was to evaluate the antioxidant and pro-inflammatory state using biomarkers in patients with MetS, according to the adherence to MDP.

## 2. Methods

### 2.1. Study Design and Participants

Ninety adult subjects (aged 55–75 years old; body mass index (BMI) between ≥27 and <40 kg/m^2^; 54% women) living in Mallorca (Spain) participated in the study. To participate in the study, patients had to meet three or more of the MetS criteria: (1) waist circumference ≥80 cm in women and ≥90 cm in men; (2) blood pressure ≥130/85 mmHg; (3) fasting serum glucose level ≥100 mg/dL; (4) HDL-cholesterol <50 mg/dL in women and <40 mg/dL in men; (5) triglycerides ≥150 mg/dL; according to the updated harmonized definition of the International Diabetes Federation, National Heart, Lung, and Blood Institute, and the American Heart Association and [24].

Participants were distributed into two groups according to the score obtained on their adherence to the MDP. The MDP was evaluated by means of a validated questionnaire, which has a score of 17 items of the Mediterranean diet [25]. Therefore, the first group was conformed of 45 participants who had low adherence to the MDP, and the other group was conformed of 45 participants who had high adherence to the MDP.

The experimental procedure was designed following the Declaration of Helsinki and was revised and approved by the Ethics Committee of Research of Balearic Islands (CEIC-IB2251/14PI). All participants were informed of the purpose and the implications of the study, and informed consent was obtained from all subjects.

### 2.2. Anthropometric Measurements

Anthropometric measurements were performed by professional dieticians who underwent identical and rigorous training in an effort to reduce the effects of inter-observer coefficients of variation. Height was determined using a mobile anthropometer (Seca 213, SECA Deutschland, Hamburg, Germany) to the proximate millimetre, with the subject’s head in the Frankfurt plane. Bodyweight and body fat was determined using a Segmental Body Composition Analyzer (Tanita BC-418, Tanita, Tokyo, Japan). The participants were weighed without shoes and light clothes, subtracting 600 g for their clothes. BMI was calculated using weight (kg)/height (m^2^).

Blood pressure was measured in triplicate, waiting for 1 min between each determination, with a validated semi-automatic oscillometer (Omron HEM, 705CP, Hoofddrop, The Netherlands) after the participant was seated for 5 min. An anthropometric tape was used to size abdominal obesity, halfway between the iliac crest and the last rib. As a cardiovascular danger biomarker, the waist-to-height ratio (WHtR) was determined from waist circumference (cm) split by height (cm). The degree of physical activity was estimated using metabolic equivalents (METs) taking into account the rate of energy waste defined according to current knowledge [26]. All the subjects described the extent of the activities completed in min/week.

### 2.3. Dietary Assessment and Dietary Inflammatory Index

A semi-quantitative 143-item food frequency questionnaire (FFQ) collected by registered dieticians and validated in Spain was used to assess dietary habits of the participants during the last 12 months [27]. Characteristic serving size was presented for each item, and the frequency of consumption was recorded in nine categories ranging from “never or hardly ever” to “≥6 times/day”.

Energy and nutrient intake was calculated by multiplying for each item the frequency by the nutrient composition of the stated serving size. This calculation was made with a computer program that integrates the information available in the food composition tables [28]. The particular frequency item was transformed into a daily intake. For all FFQ foods, the average quantity of consumed food (in grams), the average total energy intake, and macro and micronutrients were estimated. Total nutrient intake and micronutrient intake from dietary supplements reported by the participants were also considered.

The Dietary Inflammatory Index (DII) is used to assess the inflammatory potential of a diet [29]. This score derives from integrating the effect of 45 food items on six inflammatory biomarkers (IL-1β, IL-4, IL-6, IL-10, TNFα, and highly sensitive C-reactive protein (CRP)) [29]. The food values got a negative score according to whether its effect was anti-inflammatory, a positive score its effect was pro-inflammatory, and 0 if no significant change in inflammatory biomarkers was found. This index has been calculated from the dietary intake derived from the validated FFQ [30]. The values of each food parameter analyzed were subtracted from the mean value of the general database for that parameter, and then, divided by the standard deviation. The values obtained were converted into percentiles and multiplied by the specific inflammatory score of the global parameter of the food. Finally, the sum of all the specific DII scores of the food parameters provided the overall DII score. In this way, positive DII scores indicate a pro-inflammatory diet whereas negative DII scores represent a more anti-inflammatory diet.

### 2.4. Collection of Blood and Urine Samples

Blood samples were collected after 12 h overnight fasting from the antecubital vein in suitable vacutainers with and without ethylene diamine tetra acetic acid (EDTA) as an anticoagulant to obtain plasma and serum, respectively. General blood biochemical analyses were carried out on fasting serum using standard enzymatic methods in the clinical laboratory of Son Espases Hospital (Palma, Spain). Glucose, HbA1c, triglycerides, HDL-cholesterol, LDL-cholesterol, cholesterol total, and uric acid concentrations were measured in serum. Hematological parameters and hemogram were measured in an automatic flow cytometer analyser Technicon H2 VCS system (Bayer, Leverkusen, Germany).

One tube of fresh EDTA-containing blood was centrifuged at 1700× *g* for 15 min at 4 °C. An aliquot of EDTA-containing blood was also used to obtain PBMCs and neutrophils. The peripheral blood mononuclear cells (PBMCs) fraction was purified from another tube of fresh blood following a previously described procedure [31]. In brief, 6 mL of blood was poured on 4 mL of the reagent Ficoll-Paque PLUS (GE Healthcare Bio-Sciences AB, Uppsala, Sweden) and then centrifuged at 900× *g*, for 30 min at 4 °C. The PBMCs layer was recovered and washed twice with phosphate buffer saline (PBS) at pH 7.4 and centrifugated at 900× *g*, 10 min at 4 °C, one of the samples was destined for activation of cells by LPS and another sample was lysed with distilled water to measure enzyme activity. Cell lysates were stored at −80 °C until biochemical analyses. On the other hand, neutrophils were obtained from the cell precipitate using a previously described method [32]. Briefly, the precipitate of the tube, which was centrifugated at 900× *g*, 30 min at 4 °C contained the erythrocytes and neutrophils at the bottom phase. These cells were incubated at 4 °C for 30 min with 0.15 M ammonium chloride to haemolyse erythrocytes. The suspension was centrifuged at 750× *g*, for 15 min at 4 °C, and the supernatant was then discarded. The neutrophil phase at the bottom was washed first with ammonium chloride and then with PBS at pH 7.4.

Urine samples were collected in clean and dry containers on the first morning void in rest conditions. All the results of urine biochemical parameters were normalized by the levels of creatinine measured by a modified Jaffe method on an Abbott ARCHITECT c16000 (Abbott Diagnostics, Lake Bluff, IL, USA) in the clinical laboratory of Son Espases Hospital (Spain).

### 2.5. Immunoassay Kits

Interleukin-1β (IL-1β) and monocyte chemoattractant protein-1 (MCP1) levels were measured in plasma using specific ELISA kits (RayBiotech^®^, Parkway Lane, Suite, Norcross, GA, USA) according to the indicated instructions. The intra-assay coefficient of variation was intended to be lower than 10% for IL-1β and MCP1 and the calculated overall inter-assay coefficient of variation was about 12% for IL-1β and MCP1. The plasma xanthine oxidase (XOD) levels were determined using an ELISA kit (Cusabio^®^ Technology Llc, Houston, TX, USA) following supplier usage guidelines, with an intra- and inter-assay coefficients of variation of 8% and 10%, respectively. Tumour necrosis factor α (TNFα) levels were determined in plasma using an ELISA kit (Diaclone, Besancon CEDEX, France) following the manufacturer’s instructions for use. The intra- and inter-assay coefficients of variation were 3.2% and 10.9%, respectively. Ghrelin, leptin, resistin, interleukin-6 (IL-6), interleukin-15 (IL-15), and interferon-γ levels were determined in plasma using Human Custom ProcartaPlex ^TM^ (Invitrogen by Thermo Fisher Scientific, Bender MedSystems GmbH, Viena, Austria) following the supplier guidelines for use.

### 2.6. Enzymatic Determinations

Catalase activity was determined in plasma using the spectrophotometric method described by Aebi that monitors the decomposition of H_2_O_2_ [33]. SOD activity was determined following the method developed by McCord and Fridovish [34]. Myeloperoxidase (MPO) activity was measured using guaiacol as a substrate by monitoring the formation of polymerization products of oxidized guaiacol at 470 nm [35]. All the enzymatic determinations were carried out at 37 °C using a Shimadzu UV-2100 spectrophotometer (Shimadzu Corporation, Kyoto, Japan).

### 2.7. Malondialdehyde Assay

The levels of malondialdehyde (MDA) were determined in plasma and in urine of all participants by a specific colorimetric assay kit (Sigma-Aldrich Merck^®^, St. Louis, MO, USA) where the absorbance was measured at 586 nm following the manufacturer’s instructions.

### 2.8. Polyphenols Determination

Polyphenol concentration in plasma and in urine deproteinized samples with cold acetone (1:1.2) was measured by means of the Folin–Ciocalteau method [36] using a standard curve of L-tyrosine for quantification.

### 2.9. 8-Oxo-7,8-Dihydro-Guanosine, and 8-Oxo-7,8-Dihydroguanosine Determination

The urinary 8-oxo-7,8-dihydro-guanosine (8-oxodG) and 8-oxo-7,8-dihydroguanosine (8-oxoGuo) concentrations were analysed by ultra-performance liquid chromatography coupled with tandem mass spectrometry (UPLC-MS/MS; Waters, Milford, MA, USA) methodology as previously reported [37].

### 2.10. PBMCs and Neutrophils ROS Production

ROS production by PBMCs and neutrophils was measured after activation with lipopolysaccharide (LPS) from *Escherichia coli* (Sigma-Aldrich, St. Louis, MO, USA). Cell suspensions (6 × 10^5^ cells) were introduced in a 96-well microplate containing LPS prepared in 2 mM in phosphate buffer saline, pH 7.4. Then, the cell-permeant probe 2,7-dichlorofluorescein-diacetate (DCFH-DA, 61.6 μM in Hanks’ Balanced Salts Medium) as indicator was added to all wells. The fluorescence (Ex, 480 nm; Em, 530 nm) was registered for 1 h at 37 °C in FLx800 Microplate Fluorescence Reader (Biotek Instruments, Inc., Winuschi, VT, USA).

### 2.11. Statistics

All statistical analyses were carried out with the Statistical Package for Social Sciences (SPSS v.27, IBM Software Group, Chicago, IL, USA). A Kolmogorov-Smirnov test was previously applied to assess the normal distribution of the data. The student’s *t*-test for impaired data was used to determine the significance of the data. Results are expressed as mean ± standard error of the mean (SEM) and *p* < 0.05 was considered statistically significant. Correlations between adherence to the MDP and the different parameters analysed were also assessed using the Pearson correlation coefficient.

## 3. Results

**Figure 1:**
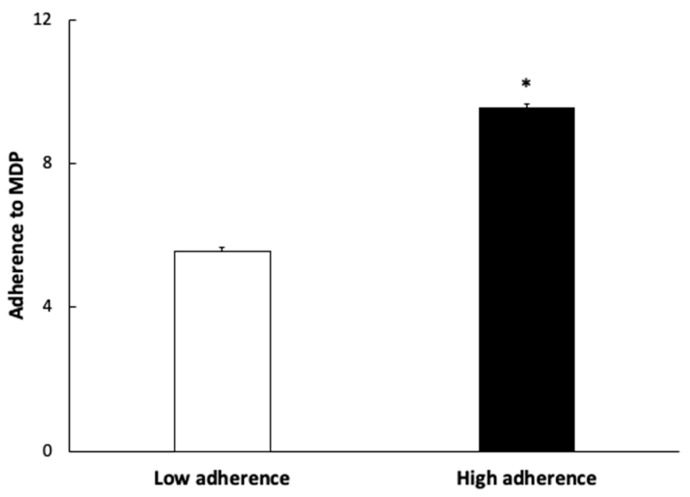
Adherence to Mediterranean Dietary Pattern (MDP) expressed as points obtained in the questionnaire. Results are presented as mean ± SEM. Data points in bold (*) are significant (*p* < 0.05) by Student *t*-test.

Figure 1 shows the values of adherence to the MDP of subjects according to their low and high adherence. The group with lower adherence to the MDP showed a mean value of 5.54 ± 0.11 and the group with higher adherence to the MDP was 9.54 ± 0.14.

The anthropometric characteristics of participants stratified by the adherence to MDP are shown in Table 1. Although the height and weight values were higher in the group with low adherence to MDP, the BMI values were similar between both groups. HbA1c, lower HDL-cholesterol, and triglycerides levels were higher in the group with low adherence to the MDP. Other parameters analyzed did not show differences between the two groups.

Table 2 shows the nutrients, vitamins, and minerals ingested by participants according to adherence to the MDP. A higher intake of trans-FA and SFA, and a lower intake of w-3 FA, fibre, and vitamin C and D were observed in the participants with lower adherence to MDP. Consequently, participants with higher adherence to MDP showed lower DII values than participants with lower adherence to MDP.

The haematological parameters of participants are shown in Table 3. No differences were evidenced in haematocrit, erythrocyte, leukocytes, neutrophils, lymphocytes, monocytes, eosinophils, and basophils counts between both groups.

The results of the ELISA and multiplex assays, MDA, polyphenols, 8oxoGuo, and 8oxodG are shown in Table 4. XOD, IL-1β, IL-6, IL-15, TNFα, and ghrelin plasma levels were higher in subjects with low adherence to the MDP, whereas no differences were found in MCP1, leptin, resistin, interferon-γ, MDA, and polyphenol levels between groups. Polyphenol urine levels normalized by the levels of creatinine were higher in subjects with high adherence to the MDP, whereas no differences were found in 8oxoGuo, 8oxodG, and plasma.

**Figure 2:**
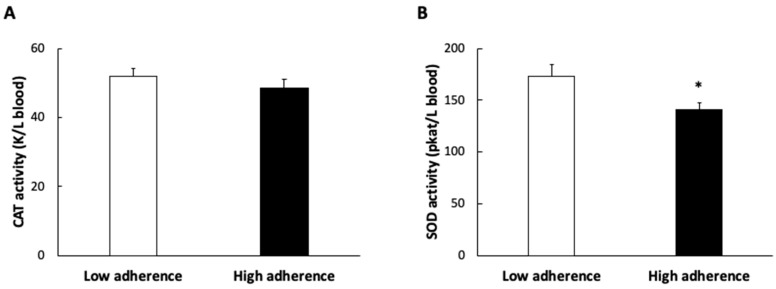
Plasma catalase (CAT) enzymatic activity (**A**) and superoxide dismutase (SOD) enzymatic activity (**B**) of patients according to the Mediterranean Dietary Pattern score. Results are presented as mean ± SEM. Data points in bold (*) are significant (*p* < 0.05) by Student *t*-test.

The activities of the antioxidant enzymes catalase and SOD are shown in Figure 2. The activity of SOD was higher in subjects with lower adherence to the MDP, but no differences were found in catalase activity between groups.

**Figure 3:**
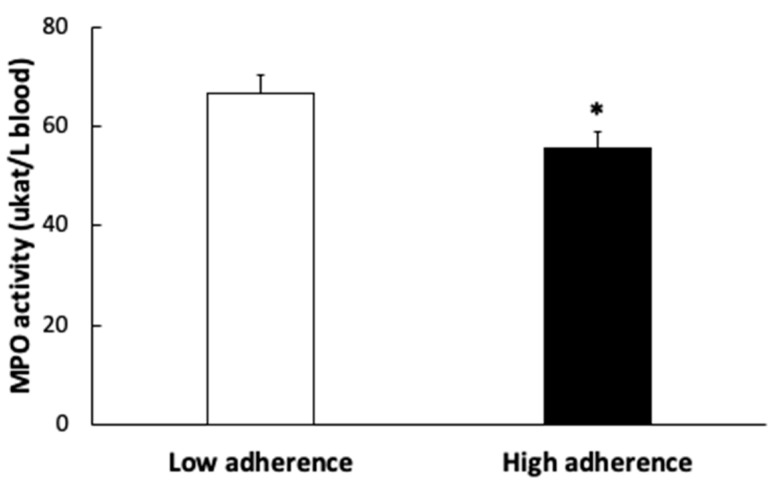
Plasma myeloperoxidase (MPO) enzymatic activity of patients according to the Mediterranean Dietary Pattern score. Results are presented as mean ± SEM. Data points in bold (*) are significant (*p* < 0.05) by Student *t*-test.

MPO activity (Figure 3) was also higher in the group with lower adherence to the MDP.

**Figure 4:**
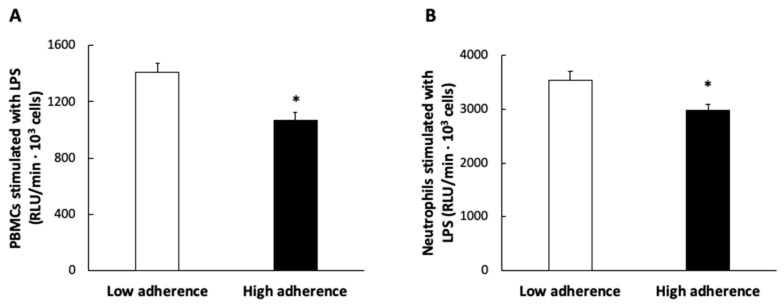
Peripheral blood mononuclear cells (PBMCs) (**A**) and neutrophils (**B**) stimulated with lipopolysaccharide (LPS) according to the Mediterranean Dietary Pattern score. Results are presented as mean ± SEM. Data points in bold (*) are significant (*p* < 0.05) by Student *t*-test.

The results of the activation of PBMCs and neutrophils by LPS classified according to the adherence to the MDP are shown in Figure 4. Both cell types showed higher response to activation with LPS in the group with lower adherence to MDP than those with higher adherence.

Table 5 shows the correlations between the different parameters analyzed and the MDP adherence score. Weight, abdominal obesity, triglycerides, and plasma ghrelin were inversely correlated with greater adherence to MDP with *p* < 0.01 and height, HbA1c, plasma IL-1β, and XOD levels, ROS production in PBMCs by LPS, and urine MDA with *p* < 0.05.

## 4. Discussion

The current results evidenced that participants with MetS reporting higher adherence to the MDP showed less altered anthropometric parameters, blood biochemical profile, and better oxidative and inflammatory status than patients with MetS and low adherence to the MDP.

Although the intake in the group which presents a high adherence to the MD is healthier and has a lower inflammatory profile, this fact is not reflected in different values in plasma polyphenols. It has been reported that circulating polyphenols are rapidly conjugated to glucuronide, sulphate, and methyl groups favouring their urinary and biliary excretion evidencing maximum levels in plasma 1–2 h after consumption [38]. The lack of plasmatic differences may be due to the rapid metabolization and urinary excretion of these polyphenols. In this sense, the concentration of polyphenols/creatinine in urine was significantly higher in patients with greater adherence to MDP. These results are in accordance with previous studies that showed an increased urinary total polyphenol excretion in patients following a nutritional intervention based on MDP with high polyphenol intake [39,40]. Moreover, it has been also reported a significant correlation between polyphenol-rich foods or beverages intake with the urinary excretion of polyphenols [41,42].

MetS patients with low adherence to the MDP showed higher body weight, levels of HbA1c and triglycerides, and lower HDL cholesterol. These patients, although not significantly, also showed higher abdominal obesity. These alterations are associated with unbalanced nutritional status and, if they are not reversed, can lead to the development of T2DM [11]. Therefore, following an MDP has been related to an improvement in glucose and lipid profile in individuals with obesity and MetS, associated with an increased intake of omega-9 fatty acids, vitamin E, zinc, and selenium, and a decrease in SFA [43]. A systematic review and meta-analysis of 41 different studies concluded that subjects reporting high adherence to MDP showed higher HDL-cholesterol concentration and lower levels of triglycerides than subjects with lower adherence to MDP [44].

Concerning blood immune cells, no differences in the haematocrit and number of circulating erythrocytes, total leukocytes as well as neutrophils, and PBMCs have been observed between both groups. These results suggest that, although MDP has protective effects against CVD and obesity [8], it does not appear to exert any significant effect on the number of circulating immune cells in patients with MetS. However, the stimulation of both neutrophils and PBMCs with LPS induces a greater capacity of these to produce ROS in the group with lower adherence to the MDP, which would indicate that the cells are in a higher degree of pre-activation and, therefore, favour a more pro-inflammatory state [45]. In a previous study, we evidenced a progressive increase in the production of ROS by PBMCs with BMI favouring oxidative stress and inflammation in obese patients compared to overweight and normal weight [45]. Moreover, it has been evidenced that the diet supplementation with blueberries, rich in antioxidants, reduces ROS production by monocytes in adults with MetS [46]. The higher pre-activation state of immune cells is complemented by higher plasma MPO activity, a marker of leukocyte activation, which may jointly contribute to a higher degree of pro-oxidative state in patients with fewer adherences to MDP. It has been shown that dietary supplementation with high quality-extra virgin olive oil, an essential component of MDP with protective effects against chronic diseases, decreased MPO activity after three months of intervention [47].

Other enzymes such as XOD can be a source of ROS in the blood vessels, which could induce inflammation and damage to tissues [48]. XOD generates a superoxide anion that may contribute to endothelial dysfunction and has been related to CVD and MetS. In fact, this enzyme has been reported to be increased in MetS subjects compared to healthy subjects, suggesting a potential role in the pro-oxidative state related to MetS [48]. In the current study, XOD levels in patients with greater adherence to the MDP are lower than in patients with lower MDP. This situation could be related to the beneficial effects of this dietary pattern on the MetS components. Similarly, in a previous study, we observed a decrease in plasma XOD after a 5-year nutritional intervention with a Mediterranean diet supplemented with extra-virgin olive oil or with nuts [49]. Moreover, our findings also evidenced that those patients with greater adherence to the MDP show the lower activity of the antioxidant enzymes CAT and SOD. These outcomes are in accordance with previous studies that reported how high levels of reactive species are capable of reducing antioxidant enzymatic activities such as CAT and SOD [50,51]. These differences could also be related to the fact that a better quality diet can increase the levels of exogenous antioxidants in plasma, in addition to being a diet with higher components with an anti-inflammatory profile [52]. In addition, levels of MDA, the product of lipid peroxidation, did not show differences according to the adherence of patients to the MDP, suggesting that the increases in antioxidant enzymes could be enough to prevent the increase in oxidative damage in these patients. These results are also supported by the absence of differences in urinary MDA but also in 8oxoGuo/creatinine and 8oxodG/creatinine usually used to determine the existence of oxidative stress affecting nucleic acids [53]. Although there are no differences in oxidative damage markers, it has been established that subjects with MetS have higher plasma levels of MDA [45]. Accordingly, it is important to work with these patients to not only improve their diet but also reduce their BMI and associated risk factors.

When analysing the circulating levels of various cytokines, the current results showed lower generalized levels in the group with the highest adherence to the MDP. Specifically, subjects with higher adherence to MDP showed lower levels of the pro-inflammatory cytokines IL-6 and TNFα than subjects with lower adherence to MDSP. These results are in accordance with previous studies demonstrating that a diet high in ω-3 FA, such as an MD promoting the consumption of virgin olive oil, nuts, and seeds, reduces IL-6 and TNFα levels by inhibiting the synthesis of these pro-inflammatory cytokines [54,55]. Lower concentrations of IL-1β were also shown in the participants who had greater adherence to MDP than those presenting lower adherence to MDP. IL-1β, like MCP-1 and INF-γ, are cytokines involved in the NF-κβ signalling pathway [56]. Although the current results did not show differences in MCP-1 and INF-γ, a previous study observed that after an intervention nutritional with an MD of 3 years, plasma levels of both cytokines significantly decreased [56]. Furthermore, the current results revealed that participants with higher adherence to the MDP show lower concentrations of IL-15, an immune cytokine implicated in autoimmune diseases that acts as a potent pro-inflammatory mediator inducing the expression of TNFα, IL-1 and INF-γ [57]. Accordingly, in a random sample of the Balearic Islands population, it was observed that low adherence to MDP is related to higher levels of plasmatic inflammation markers, including TNF-α, and plasminogen activator inhibitor 1 (PAI-1) and CRP [58]. It has been seen how weight loss following a Mediterranean diet is directly related to the reduction of the levels of inflammatory cytokines [59]. In fact, it has been suggested that some cytokines are produced by adipocytes in visceral adipose tissue as their production is reduced when there is a reduction in the waist [60].

Regarding the analysed hormones involved in the coordination of energy balance and weight regulation, differences were only observed in the case of ghrelin. Patients with higher adherence to MDP showed lower concentrations of ghrelin in plasma. This hormone, secreted by endocrine cells in the gastrointestinal tract, is associated with the control of feeding behaviour [61]. The traditional MDP is considered a satiating and tasty diet, which could contribute to the decrease in ghrelin levels in the group with higher adherence to MDP [62]. It has been also reported that ghrelin is involved in the regulation of the PBMC's response to nutrient intake and could contribute to explaining the reducing inflammatory response produced by diets enriched with PUFA ω-3 [63]. On the contrary, no differences were observed in resistin and leptin levels according to the adherence to MDP. Resistin is considered an important pro-inflammatory mediator and may play a role in the instauration insulin resistance through actions antagonistic to those of insulin [64]. It has been also reported that resistin expression in PBMCs is an important source of this hormone and its expression is induced by pro-inflammatory cytokines (TNFα, IL-6, and IL-1) [65]. Moreover, in a general population including 6637 adults, resistin levels were found to be inversely associated with adherence to the Mediterranean diet [66]. However, as all patients from the present study have metabolic syndrome characterized by a chronic pro-inflammatory state, differences in the degree of adherence to MDP may not be enough to reflect changes in resistin levels. In addition, a higher but not significant concentration of leptin was observed in the participants with higher adherence to MDP compared to those who have a lower one. The current results are in accordance with other authors who determined that following an MD does not have a significant impact on leptin levels and the ratio of leptin-to-adiponectin [67].

When analysing the existence of correlations between the degree of adherence to the MDP and the different parameters, the existence of an inverse correlation with a large part of these parameters is found. Specifically, greater adherence is associated with a better anthropometric profile, more normal blood biochemical parameters, and a lower pro-inflammatory and pro-oxidative state. In accordance, a higher adherence to MDP has been found to be associated with reduced risk of becoming overweight/obese, better insulin sensitivity and lipid profile, and reduced inflammation markers [68,69].

Although women seem to be more protected than men against cardiometabolic risk factors, the prevalence of metabolic syndrome is similar or even higher in postmenopausal women compared to that in men at a similar age [70]. In this sense, the women in the study are all postmenopausal, thus the reduction in estrogen levels favors a greater predisposition to insulin resistance and a pro-atherogenic lipid profile, minimizing the differences between genders [71]. Moreover, although the effect of gender cannot be ruled out, the presence of pro-inflammatory mediators and reactive species as factors contributing to dysfunctional metabolism arise due to a complex combination of genetic predisposition, high caloric intake, and sedentary behavior [72].

### Strengths and Limitations of the Study

The main strength of the current study is the association between high adherence to MDP and lower oxidative stress and pro-inflammatory state in patients with MetS. The main limitation of this study is that the sample size was relatively small; nevertheless, the sample size was enough to evidence the existence of differences in the biomarker levels between the group with a greater MDP and the group with lower MDP. Also, the analysis by gender could be interesting; however, the limited number of participants may make interpretation of the statistical results difficult.

## 5. Conclusions

Patients with MetS and higher adherence to the MDP showed less altered anthropometric parameters, blood biochemical profile, and better oxidative and inflammatory status than patients with MetS and low adherence to the MDP. Specifically, higher adherence was related to a lower weight, HbA1c, triglycerides, HDL-cholesterol, IL-1β, IL-6, IL-15, TNFα, XOD, and ghrelin levels, SOD and MPO activities, and reduced ROS production by PBMCs and neutrophils. Further longitudinal and long-term studies would be necessary to determine the beneficial effects of this pro-inflammatory state derived from increasing adherence to MDP.

## Figures and Tables

**Table 1:** Characteristics of participants according to adherence to median values of adherence to the Mediterranean diet pattern.

Sociodemographic and Clinical Characteristics	Reference Values	Low Adherence *n* = 45 Mean ± SEM	High Adherence *n* = 45 Mean ± SEM	* *p*-Value
Age (years)		64.2 ± 0.4	65.3 ± 0.4	0.074
Weight (kg)		89.3 ± 1.1	85.9 ± 1.2	0.036
Height (cm)		164.4 ± 0.8	161.0 ± 0.8	0.002
BMI (kg/m^2^)		33.0 ± 0.3	32.9 ± 0.3	0.999
WHtR		0.684 ± 0.005	0.683 ± 0.005	0.996
Abdominal obesity (cm)		112.3 ± 0.8	110.1 ± 0.9	0.065
Systolic blood pressure (mmHg)	<130	143.1 ± 1.4	140.6 ± 1.6	0.115
Diastolic blood pressure (mmHg)	<85	83.1 ± 0.8	81.9 ± 0.8	0.111
Glucose (mg/dL)	70–110	122.6 ± 3.8	113.3 ± 1.9	0.401
HbA1c (%)	3.8–6.2	6.44 ± 0.12	6.06 ± 0.07	0.048
Triglycerides (mg/dL)	<149	158.7 ± 6.2	137.9 ± 5.2	0.021
HDL-cholesterol (mg/dL)	≥60	43.4 ± 0.9	45.3 ± 0.9	0.036
LDL-cholesterol (mg/dL)	<100	109.3 ± 3.0	113.3 ± 3.0	0.470
Cholesterol total (mg/dL)	<200	184.4 ± 3.2	186.3 ± 3.4	0.729
Uric acid (mg/dL)	3.5–7.2	6.24 ± 0.13	6.22 ± 0.10	0.808
Total physical activity (MET·min/week)		2972 ± 278	3027 ± 211	0.080

Abbreviations: BMI: body mass index; HbA1c: glycated haemoglobin A1c, MET: metabolic equivalent of task, SEM: standard error media; WHtR: waist-to-height ratio. Results are expressed as mean ± SEM. * *p*-values by Student’s *t*-test for impaired data.

**Table 2:** Nutrients, vitamins, and minerals ingested by participants according to median values of adherence to the Mediterranean Diet Pattern.

	Low Adherence *n* = 45 Mean ± SEM	High Adherence *n* = 45 Mean ± SEM	* *p*-Value
**Nutrients**			
Trans FA (g/day)	0.759 ± 0.042	0.592 ± 0.033	0.002
w-3 FA (g/day)	0.694 ± 0.034	0.885 ± 0.038	<0.001
SFA (g/day)	29.2 ± 1.0	26.2 ± 0.8	0.017
MUFA (g/day)	52.5 ± 1.5	53.8 ± 1.6	0.584
PUFA (g/day)	18.0 ± 0.6	18.2 ± 0.7	0.892
Cholesterol (mg/day)	411.3 ± 12.8	379.3 ± 10.3	0.053
Fibre (g/day)	23.4 ± 0.8	29.6 ± 0.9	<0.001
**Vitamins**			
Vitamin A (μg/day)	1166.5 ± 56.8	1255.1 ± 57.4	0.273
Vitamin C (mg/day)	188.5 ± 7.0	221.6 ± 8.5	0.003
Vitamin D (μg/day)	4.95 ± 0.25	6.13± 0.29	0.002
Vitamin E (mg/day)	10.9 ± 0.4	11.2 ± 0.36	0.511
**Minerals**			
Selenium (μg/day)	108.6 ± 3.2	114.5 ± 3.0	0.179
Zinc (mg/day)	12.9 ± 0.3	13.5 ± 0.4	0.233
**Dietary Indices**			
DII	0.951 ± 0.152	−0.400 ± 0.175	<0.001

Abbreviations: DAI: dietary antioxidant index; DII: dietary inflammatory index; MUFA: monounsaturated fatty acid; PUFA: polyunsaturated fatty acid; SEM: standard error media; SFA: saturated fatty acid; trans FA: trans-fatty acid; ω-3 FA: omega-3 fatty acid. Results are expressed as mean ± SEM. * *p*-values by Student’s *t*-test for impaired data.

**Table 3:** Haematological parameters of participants according to median values of adherence to the Mediterranean Diet Pattern.

	Reference Values	Low Adherence *n* = 45 Mean ± SEM	High Adherence *n* = 45 Mean ± SEM	* *p*-Value
Haematocrit (%)	40.0–50.0	42.9 ± 0.5	42.7 ± 0.3	0.323
Erythrocytes (10^6^/mm^3^)	4.5–5.8	4.83 ± 0.04	4.74 ± 0.04	0.133
Leukocytes (10^3^/mm^3^)	4.0–11.0	7.58 ± 0.17	7.27 ± 0.17	0.197
Neutrophils (10^3^/mm^3^)	1.8–7.5	4.77 ± 0.41	4.01 ± 0.14	0.145
Lymphocytes (10^3^/mm^3^)	1.0–4.5	2.63 ± 0.20	2.38 ± 0.07	0.463
Monocytes (10^3^/mm^3^)	0.0–1.0	0.744 ± 0.074	0.674 ± 0.055	0.303
Eosinophils (10^3^/mm^3^)	0.0–0.5	0.264 ± 0.034	0.273 ± 0.045	0.277
Basophils (10^3^/mm^3^)	0.0–0.2	0.057 ± 0.008	0.085 ± 0.032	0.339

Abbreviations: SEM: standard error media. Results are expressed as mean ± SEM. * *p*-values by Student’s *t*-test for impaired data.

**Table 4:** Oxidative stress and inflammatory plasma markers of participants according to the median values of adherence Mediterranean Diet Pattern.

	Low Adherence *n* = 45	High Adherence *n* = 45	* *p*-Value
	Mean ± SEM	Mean ± SEM	
**Protein levels**			
XOD (ng/mL)	0.444 ± 0.024	0.336 ± 0.021	<0.001
IL-1β (pg/mL)	10.3 ± 0.5	8.33 ± 0.38	0.035
IL-6 (pg/mL)	6.71 ± 0.71	3.09 ± 0.41	0.001
IL-15 (pg/mL)	8.98 ± 0.44	5.98 ± 0.66	0.012
TNFα (pg/mL)	67.8 ± 4.4	56.3 ± 2.9	0.015
Interferon-γ (pg/mL)	5.91 ± 0.57	5.47 ± 0.40	0.316
MCP-1 (pg/mL)	259.1 ± 12.8	244.5 ± 35.7	0.063
Resistin (ng/mL)	5.45 ± 0.92	4.91 ± 0.78	0.643
Ghrelin (pg/mL)	327.2 ± 11.5	271.3 ± 8.6	0.002
Leptin (ng/mL)	10.4 ± 1.1	13.2 ± 2.8	0.631
**Plasma markers**			
MDA (nM)	0.922 ± 0.071	0.928 ± 0.066	0.821
Polyphenols (nM)	0.058 ± 0.002	0.057 ± 0.002	0.392
**Urine markers**			
8-oxoGuo/Creatinine (nM/mM)	2.01 ± 0.05	1.93 ± 0.06	0.244
8-oxodG/Creatinine (nM/mM)	1.47 ± 0.06	1.39 ± 0.06	0.509
MDA/Creatinine (mM/mM)	102.0 ± 9.2	88.0 ± 7.5	0.159
Polyphenols/Creatinine (mM/mM)	13.6 ± 1.0	16.0 ± 1.1	0.008

Abbreviations: IL-1β: interleukin-1β; IL-6: interleukin-6; IL-15: interleukin-15; INF-γ: interferon-γ; MCP-1: monocyte chemoattractant protein 1; MDA: malondialdehyde; 8-oxoGuo: 8-oxo-7,8-dihydroguanosine; 8-oxodG: 8-oxo-7,8-dihydro-2′-deoxyguanosine; SEM: Standard error media; TNFα: tumour necrosis factor α; XOD: xanthine oxidase. Values are expressed as mean ± SEM. * *p*-values by Student’s *t*-test for impaired data.

**Table 5:** Correlation between adherence to the Mediterranean Diet Pattern and parameters analyzed.

	Correlation
Weight	−0.178 **
Height	−0.182 *
Abdominal obesity	−0.173 **
HbA1c	−0.137 *
Triglycerides	−0.197 **
Plasma IL-1β	−0.230 *
Plasma XOD	−0.228 *
Plasma Ghrelin	−0.319 **
ROS production in PBMCs by LPS	−0.220 *
Urine MDA	−0.165 *

Abbreviations: HbA1c: Glycated haemoglobin A1c; IL-1β: interleukin-1; LPS: lipopolysaccharide; MDA: Malondialdehydeβ; PBMCs: peripheral blood mononuclear cells; ROS: reactive oxygen species; XOD: xanthine oxidase. Bivariate correlation: * *p* < 0.05; ** *p* < 0.01.

## Data Availability

There are restrictions on the availability of data for this trial, due to the signed consent agreements around data sharing, which only allow access to external researchers for studies following the project purposes. Requestors wishing to access the trial data used in this study can make a request to the corresponding author.
